# Towards a new model of population mental health research and policy translation in the UK: establishing a National consortium

**DOI:** 10.1186/s13033-026-00703-2

**Published:** 2026-05-02

**Authors:** Tassia Kate Oswald, Joseph Barker, Daniel Barrett, Ruth Blackburn, Erica Breuer, Natasha Chilman, Natasha Cutler, Helen Daly, Niamh Doherty, Abd Doumany, Johnny Downs, Alexandru Dregan, Rina Dutta, Jacqueline Dyer, Laura Fischer, Stephani L. Hatch, Sumaty Hernandez, Matthew Hotopf, Annie Jeffery, Ann John, James B. Kirkbride, Lee Knifton, Caglar Koksal, Gerard Leavey, Ngozi Oparah, Isaac Ouro-Gnao, Lisa Marzano, Paul Patterson, Rebecca Rhead, Peter Schofield, Kalwant Sidhu, Sarah Steeg, Sharon Stevelink, Matt Sutton, Roger T. Webb, Jenny Woodman, Jayati Das-Munshi

**Affiliations:** 1 Department of Psychological Medicine, Institute of Psychiatry, Psychology and Neuroscience, King’s College London, 16 De Crespigny Park, London, SE5 8AB UK; 2 Population Health Improvement UK (PHIUK), Cambridge, UK; 3 Department of Psychological Medicine, Institute of Psychiatry, Psychology & Neuroscience, King’s College London, London, UK; 4 Centre for Mental Health & Community Wellbeing, Melbourne School of Population & Global Health, The University of Melbourne, Melbourne, Australia; 5 Thrive LDN, London, UK; 6 UCL Great Ormond Street Institute of Child Health, University College London, London, UK; 7 School of Medicine and Public Health, The University of Newcastle, Newcastle, Australia; 8 Department of Population Health Sciences, Faculty of Life Sciences and Medicine, King’s College London, London, UK; 9 ESRC KCL Centre for Society and Mental Health, London, UK; 10 Bamford Centre for Mental Health & Wellbeing, Ulster University, Coleraine, UK; 11 Traumascapes, CIC, London, UK; 12 Department of Child & Adolescent Psychiatry, Institute of Psychiatry, Psychology & Neuroscience, King’s College London, London, UK; 13 South London and Maudsley NHS Foundation Trust, London, UK; 14 Black Thrive Global, Lambeth, UK; 15 Division of Psychiatry, University College London, London, UK; 16 Health Data Science, Swansea University Medical School, Swansea University, Swansea, Wales, UK; 17 Mental Health Foundation, London, UK; 18 Department of Planning, Property and Environmental Management, The University of Manchester, Manchester, UK; 19 Department of Psychology, Faculty of Science and Technology, Middlesex University, London, UK; 20 Forward Thinking Birmingham, Birmingham, UK; 21 Centre for Mental Health and Safety, Division of Psychology and Mental Health, School of Health Sciences, University of Manchester, Manchester, UK; 22 Division of Population Health, Health Services Research & Primary Care, The University of Manchester, Manchester, UK; 23 Division of Psychology and Mental Health, The University of Manchester, Manchester, UK; 24 UCL Social Research Institute, University College London, London, UK

**Keywords:** Population Mental Health, Consortium, Partnerships, Data, Policy, Equity, UK

## Abstract

**Background:**

Mental health conditions account for 18% of years lived with disability worldwide. 1-in-6 adults are affected in England, with most mental health conditions beginning in childhood and adolescence. Mental distress and ill health are unequally distributed in the UK, with strong associations with wider determinants of health, and higher prevalence among systemically disadvantaged groups. Currently, there is a lack of evidence to inform effective and timely policymaking for primary prevention in the UK.

**Methods:**

In recognition of these challenges, a national Population Mental Health (PMH) Consortium was established, as part of Population Health Improvement UK (PHIUK). PHIUK is a national research network which works to transform health and reduce inequalities through change at the population level. Our aim is to establish an interdisciplinary PMH Consortium, focussing on upstream determinants and the prevention of risks and onset of mental health conditions through interdisciplinary stakeholder engagement, to create new opportunities for population-based improvement of mental health in the UK.The PMH Consortium brings together leading interdisciplinary representation in population mental health, spanning from sciences to the arts, across the UK. Membership includes six academic institutions, third sector organisations, lived experience expertise, and strong links with national bodies to ensure integrated cross-national and regional policy impact. The PMH Consortium comprises four cross-cutting platforms (Partners in policy, implementation, and lived experience; Data, linkages, and causal inference; Narrowing inequalities; Training and capacity building) and three challenge areas (Children and young people’s mental health; Prevention of suicide and self-harm; Multiple long-term conditions) which are highly integrated and interdependent. The work will be underpinned by a Theory of Change across an initial four-year life cycle.

**Conclusion:**

This paper describes the aim, objectives, and approach of the PMH Consortium, as well as anticipated challenges and strengths. The goal of the PMH Consortium is to develop a model for population mental health research and policy translation that is both scalable and sustainable. It is critical to ensure continued impact and viability beyond the initial four years, contributing to the prevention of mental health conditions in the UK, with personal, economic, social, and health benefits.

**Supplementary Information:**

The online version contains supplementary material available at 10.1186/s13033-026-00703-2.

## Background

Globally, mental health conditions are in the top ten leading causes of disability [1], accounting for almost 18% of years lived with disability, globally. In England, 1-in-6 adults have a common mental health condition, and less than 40% of these individuals access treatments [2]. Mental distress and ill-health are unequally distributed, with strong associations noted with determinants of health and higher prevalence among systemically disadvantaged groups [3, 4], leading to significant disability, difficulty fulfilling educational and professional roles, sickness absence, and wide-ranging adverse social and economic impacts across the lifespan [1, 5]. Despite this, there is a lack of evidence to inform policymaking for primary prevention, with only 4% of UK mental health research focussed on prevention [6].

Historically, mental health has been viewed as residing outside of public health because major public health efforts and decisions have generally been informed by mortality statistics, with less emphasis placed on morbidity [7]. This undervalues the significant global contribution of mental distress and ill health [1]. The historic separation of physical and mental health in the Global North, in part due to systemic stigma associated with mental illness [1], has further contributed to an absence of effective population strategies to prevent the onset of mental health issues, as well as continuing lack of parity of esteem between mental and physical healthcare [8]. Challenges around translation of evidence into implementation and policy change further hinder improvements in population mental health. For example, a 2011 study reported that on average there was an estimated 17-year lag for published translational health research evidence to be implemented in practice (e.g., across all health conditions, including clinical, pharmacological, and healthcare guidelines) [9], by which time evidence may be outdated in rapidly changing societies.

Effective population interventions, targeting the broader social and structural determinants of mental distress and ill health, are urgently needed and could have profound benefits for society. Mental health is underpinned by a complex system of interdependent factors [10]; therefore, to produce the greatest benefit, prevention initiatives will need to transverse the siloed nature of current approaches [11], which have tended to use ‘single disease framework’ approaches [12]. This approach has major limitations for mental health, which frequently co-occurs with other health conditions [3, 6]. Approaches to population mental health will only be effective if driven by both health and non-health-related sectors that address known social and structural determinants of mental health, such as education, social care, and urban planning. Approaches must also embed a broad range of interdisciplinary stakeholders, including people with lived experience, policymakers, and public health practitioners. Moreover, participation must be inclusive of diverse communities to ensure that the right questions are asked by the right people, with findings rapidly translated into actionable and tangible recommendations. Specifically, co-design approaches are particularly important in mental health research, as they help to address long-standing power imbalances, centre lived experience alongside professional expertise, and improve the relevance, acceptability, and impact of research, interventions, and policies [13].

### The Population Health Improvement UK (PHIUK) Population Mental Health (PMH) Consortium

In recognition of these challenges, a national Population Mental Health (PMH) Consortium was established, as part of Population Health Improvement UK (PHIUK). PHIUK is a national research network which works to transform health and reduce inequalities through change at the population level. The aim of the PMH Consortium is to establish an interdisciplinary and distributed Population Mental Health (PMH) Consortium, which is focussed on upstream determinants and the prevention of risks and onset of mental health issues through interdisciplinary stakeholder engagement, to create new opportunities for population-based improvement of mental health in the UK. As far as we are aware, this is the only population mental health consortium to be established with these explicit aims and with the specific methodologies towards inclusive stakeholder and lived experience involvement. Therefore, in this paper we set out our approaches to inform the field, in this growing area.

## Methods

The PMH Consortium will establish an ‘evidence-to-policy’ and ‘policy-to-evidence’ pipeline, ensuring impact on population mental health, by addressing social determinants and identifying effective upstream interventions, to shift the focus toward prevention over reactive treatment [3, 4, 14]. We will facilitate dialogue enabling identification, prioritisation, and evaluation of public health and policy interventions locally, regionally, and nationally, leveraging insights from lived experience and large-scale UK linked data, underpinned by the application of novel, rigorous methods, capacity building, and involvement of the public and local communities. Related to this protocol, Table S1 in the Supplementary Materials presents a GRIPP2 reporting checklist of patient and public involvement in research.

### Research setting

The PMH Consortium is jointly directed by King’s College London and Thrive LDN (a public mental health partnership, with local government, across Greater London), underscoring our commitment to meaningful partnerships that ensure research insights are translated effectively, and in a timely way, into real-world impact. Membership includes six academic institutions (King’s College London, University College London, Middlesex University, The University of Manchester, Ulster University (Northern Ireland), and Swansea University (Wales)) and third sector organisations, including expertise in lived experience, survivor research, community support, and creative methods (Thrive LDN, Traumascapes, Mental Health Foundation (including Scotland), Black Thrive Global, Forward Thinking Birmingham), with strong links to national bodies representing health and the voluntary sector, in the UK (Office for Health Improvement and Disparities, Association of Directors of Public Health, Centre for Mental Health, NHS Race and Health Observatory) across England, Scotland, Wales, and Northern Ireland, ensuring integrated cross-national and regional policy impact of the research.

### Status

**Fig. 1:**
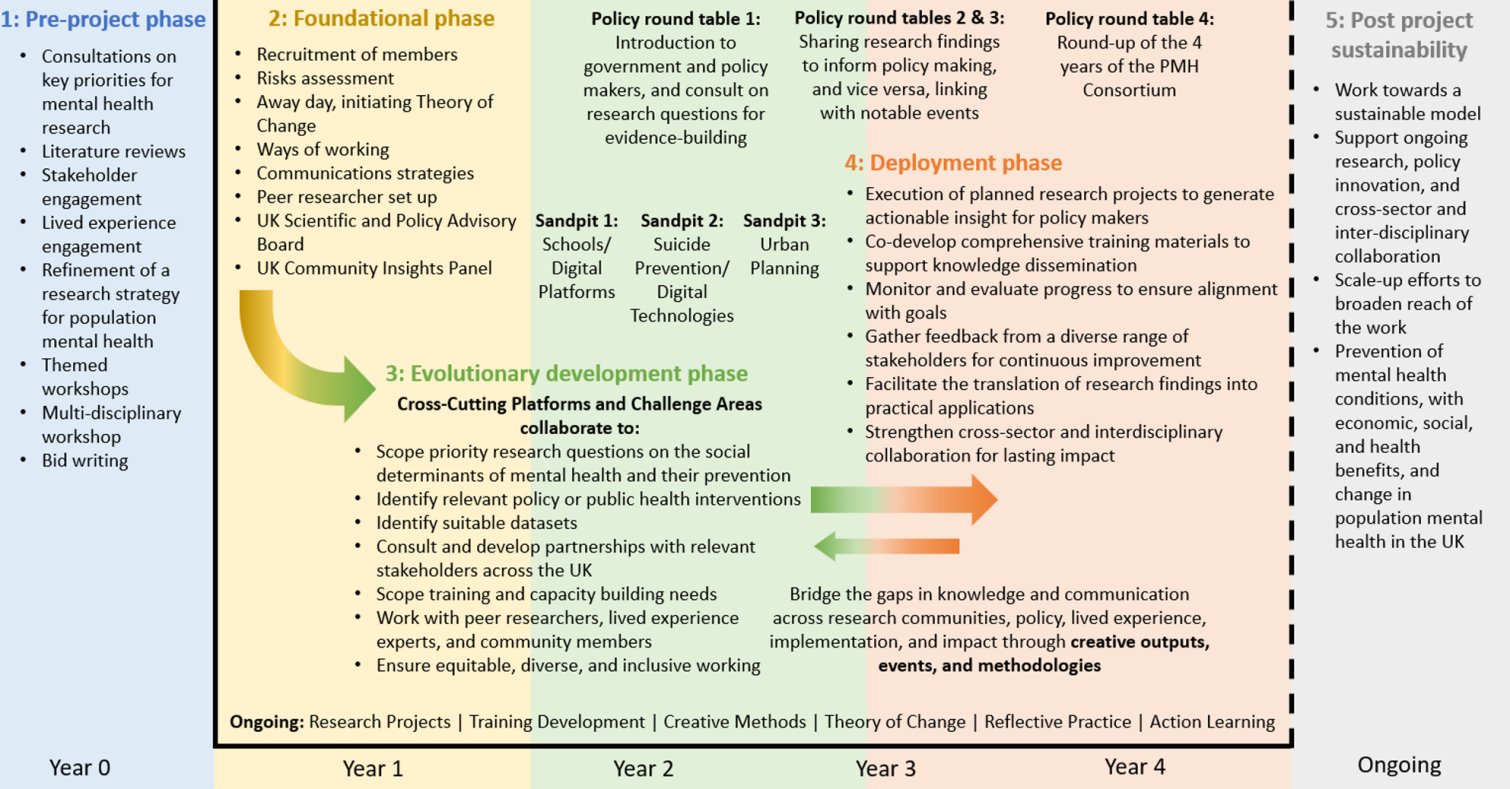
The Population Mental Health (PMH) Consortium Life Cycle

The PMH Consortium started on 01 April 2024. As shown in Fig. 1, the PMH Consortium life cycle involves five key phases: pre-project, foundational, evolutionary development, deployment, and post-project sustainability. At the time of preparing this paper, we had completed the pre-project phase and were in the foundational phase.

### Theoretical underpinning of the PMH Consortium

Theory of Change is a planning and evaluation method, often developed through workshops with relevant stakeholders, which has been increasingly used across public health programmes and interventions to develop and describe the path to their intended impact [15–18]. We commenced development of a Theory of Change during the pre-project phase, which involved evidence reviews and consultation with diverse stakeholders. In the foundational phase, we further developed the Theory of Change through a PMH Consortium Away Day, in which we collectively identified population mental health needs and our assets (see Table 1). Forty-five individuals, from 10 + organisations/sectors, attended the away day and contributed to this work. The Theory of Change will be flexibly and iteratively developed across the foundational and evolutionary development phase, to guide the PMH Consortium towards a sustainable and scalable model which creates new and ongoing opportunities for population-based improvement of mental health in the UK.

**Table 1:** Collectively identified needs and assets

Population Mental Health Needs		PMH Consortium Assets
**Trust in Communities**: Building trust within the communities the consortium serves is essential for improving outcomes and creating lasting impact.	**⇨**	**Lived Experience Expertise**: Access to experts with lived experience engagement will provide invaluable insights on community engagement.
**Breaking Silos**: Collaboration across different disciplines must be enhanced to avoid fragmented approaches and to ensure that everyone works toward shared goals.	**⇨**	**Diverse Expertise**,** Knowledge and Understanding**: The consortium benefits from diverse expertise, methodologies, and perspectives of different groups.
**Knowledge Equity**: There is a need for blending different types of knowledge (e.g., academic, lived experience) in a way that avoids hierarchies and respects all contribution.	**⇨﻿**	**Collaborative Energy**: There is a shared commitment to building a unified and energising approach that will strengthen the work of the consortium.
**Expanding Knowledge Horizons**: A need to explore what data and policies exist elsewhere e.g., internationally, to inform approaches.	**⇨﻿**	**Geographical Spread**: Experiences from UK devolved nations contribute to a broader understanding of challenges and solutions.
**Transparent Data**: Public-friendly and transparent ways of presenting data to ensure that it is accessible and understandable to all stakeholders, particularly those from the communities involved.	**⇨﻿**	**Data Access and Interpretation**: Members have access to diverse datasets and the expertise to analyse and critique them effectively. Data presentation and dissemination will be co-designed with people with lived experience.
**Policy Translation**: Evidence needs to be effectively translated into policies that decision-makers can implement, bridging the gap between research and practice.	**⇨**	**Blend of Leadership**: The collaboration between academic, third sector, and national bodies in leadership roles ensures that decisions are grounded in practical experience and incorporate community focus.

### Ethical, effective, and equitable ways of working: Centring lived experience and ensuring equality, diversity, and inclusion

Perspectives drawn from lived experience provide retrospective insights not only on mental health issues but also on risk, protective, and preventative factors – providing key insights on what should be considered for prevention of mental ill-health. To understand and address mental health issues and their bidirectional relationship with inequalities, we must understand these from the perspectives of those most affected and ensure that a diverse range of experiences are considered.

Additionally, oppression is enacted through structures and social norms, but these are upheld by people. Though not confined to individual actions, oppression is sustained – and dismantled – through the beliefs we hold and the decisions and actions we take based on these; therefore, it is critical that all Consortium members commit to anti-oppressive practice and to upholding principles of equity, diversity, and inclusion (EDI).

PMH Consortium members recognise that meaningful progress for population mental health requires a commitment to Equity, Equality, Diversity, and Inclusion, ensuring our work actively challenges disparities, and advances fairer mental health outcomes for all. In doing this, we must acknowledge our roles and positionality within dynamics such as relationships with each other, external stakeholders, and the populations to whom we are accountable, while actively naming and confronting the systems of oppression in which we are embedded – such as racism, sexism, classism, ableism, homophobia, and transphobia – and the various intersections of these. By doing so, we commit to dismantling the ideas, assumptions, biases, and behaviours shaped by these systems and structures. Equality Impact Assessments provide a structured approach for doing this.

Lived experience and EDI are not complementary to population mental health but intrinsic to it; systemically disadvantaged groups are at increased risk of being subjected to violence and experiencing trauma and mental distress, therefore it is essential that those most affected are not only included in the PMH Consortium but central to its governance, leadership, and operationalisation – and that the structures and cultures of the PMH Consortium are built to support safe and meaningful engagement.

To support this, we apply trauma-informed practice principles which help us ensure our methodologies of research, engagement, and outputs centre lived experience and move from hierarchy to agency, extraction to co-creation, and control to collaboration. Trauma-informed practice refers to an approach that recognises and responds to the impact of trauma on individuals. It involves integrating an understanding of trauma’s effects into various aspects of care, interaction, and decision-making processes.

The PMH Consortium deliberately moves away from a linear view of participation and instead adopts a systemic approach in which lived and living experience meaningfully shapes the work across multiple domains and decision points. Lived experience is not confined to any one stage or part of the Consortium or conceptualised as a discrete constituency to be counted, but as a form of expertise that is embedded across multiple levels of the consortium, including leadership and co-leadership roles, governance and advisory functions, strategic decision-making, and delivery. Therefore, lived experience is resourced and distributed across individuals and structures rather than concentrated in a single group. For example, lived experience is reflected at the Co-Director and Platform Co-lead levels. In addition, specific lived and living experience roles in the consortium include: Survivor-Artist Researchers (survivor researchers who work with arts-based and creative methods), a youth advisory group, and a Community Insights Panel, which is an advisory body of 10–15 people whose members’ experiences align with the Consortium’s definition of Lived Experience (see Table S2 and Table S3 in the Supplementary Materials for definitions of lived experience and survivor research methodologies in the PMH Consortium). The Community Insights Panel’s role is to provide advice, guidance, and constructive feedback to the Consortium. The PMH Consortium is committed to reimbursing expenses and appropriately remunerating individuals who contribute their time and expertise outside of salaried roles.

Supporting active and inclusive participation is central to our vision for improving mental health and wellbeing and reducing health inequalities across all aspects of our work. To operationalise these commitments, the Consortium will use Equality Impact Assessments as a core mechanism for embedding anti-oppressive, inclusive, and trauma-informed principles across its work. Generally considered mandatory in the UK public sector, Equality Impact Assessments are a structured process for examining how a policy, project, or decision may affect different groups of people, particularly those who are marginalised or protected under UK equality law, helping to identify and address potential inequalities so that actions promote fairness and equity from the outset. Typically completed using a short assessment template or checklist by research teams (for examples see: [19–21]), these assessments will be applied at key stages of project design, delivery, and dissemination, to consider how power, privilege, and structural disadvantage may shape participation, experiences, and outcomes. They will support critical reflection on who is involved, whose voices are prioritised, and where risks of harm, exclusion, or re-traumatisation may arise, enabling the Consortium to adapt its approaches accordingly. For example, this may involve modifying recruitment strategies, providing translated materials, adjusting study procedures, or introducing additional safeguards to support safe and inclusive participation. Informed by ongoing dialogue with lived-experience partners and community collaborators, this process will help the PMH Consortium identify and respond to structural forms of inequality and discrimination, including racism and other intersecting oppressions, that influence population mental health. Failing to centre these considerations risks reinforcing a one-size-fits-all model that neglects the lived realities of many communities.

### PMH consortium cross-cutting platforms and challenge areas

**Fig. 2:**
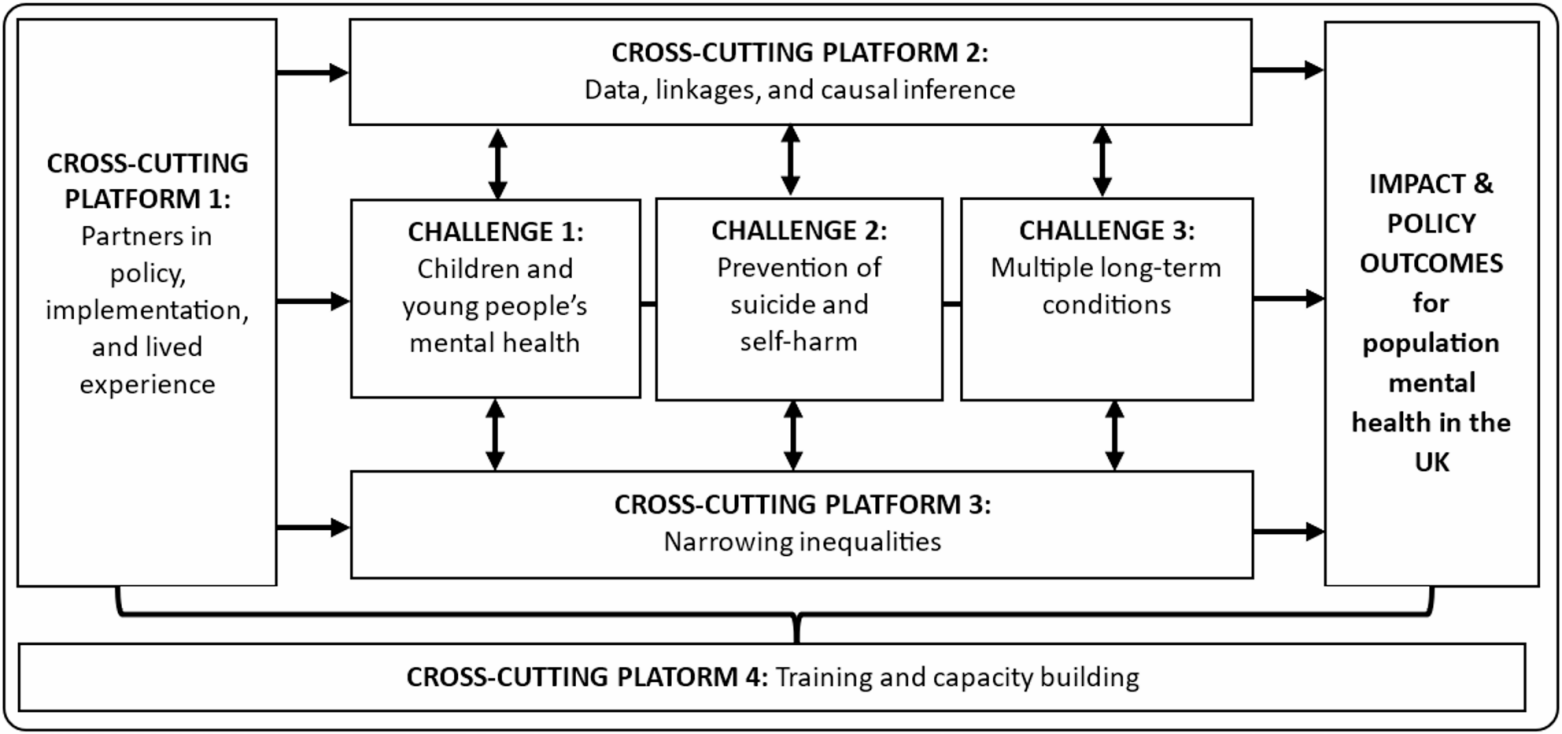
The Population Mental Health (PMH) Consortium Framework

The PMH Consortium framework comprises four Cross-Cutting Platforms and three Challenge Areas which are highly integrated and interdependent (see Fig. 2). These Cross-Cutting Platforms and Challenge Areas were selected during the pre-project phase. The framework was informed by prior work undertaken by members of the PMH Consortium in various roles, including a previously published consultation on the key priorities for mental health research into the 2020 s [22], which outlined a range of research areas which we propose to build on through our PMH Consortium; a conceptual framework for public mental health [23]; and two of the largest and most comprehensive reviews of interventions targeting the social determinants of population mental health [3, 4]. A previous strategy for population mental health (available from the authors on request) was developed and refined and formed the basis for a series of themed workshops, which included people with lived experience, and further informed the present PMH Consortium framework. Subsequent workshops included representation from UK government health bodies, regional public health representation, commissioners and voluntary sector partners; these included the Office for Health Improvements and Disparities, Department of Health and Social Care, The City Intelligence Unit (Greater London Authority), Transformation Partners in Health and Care, Toynbee Hall, Integrated Care Boards, the London Association of Directors of Public Health, and borough public health teams. The main objective, rationale, approach, and key outputs for each Cross-Cutting Platform and Challenge Area are described below.

#### Cross-Cutting Platform 1: Partners in policy, implementation, and lived experience

*Objective.* To build a coalition across government, public sector agencies, voluntary organisations, people with lived experience, and academic stakeholders, to innovate creative methods for practice-informed evidence synthesis and policy-driven research, both centred on lived experience.

*Rationale.* The disconnection between academic research, public policy, practice, and lived experience is a significant limitation for improving the mental health of all members of society. The lack of effective communication and integration across these interdependent groups has led to siloed systems and structures which perpetuate inequality and restrict improvement for health outcomes.

*Approach.* This Cross-Cutting Platform will create an infrastructure by establishing a coalition between government, public health, voluntary organisations, interdisciplinary academic stakeholders, and people with lived experience who have historically been neglected and missing from narratives. An agile project management methodology will be adopted as its principles and values challenge traditional hierarchical and top-down management structures (e.g. empowerment and collaboration, continuous improvement, open communication) [24]. We will use a range of participatory methods to inform:

1. How research and lived experience shapes policies;
2. How policy can be improved based on evidence and real-world applications and experiences;
3. Translation of findings into actions.

Cross-Cutting Platform 1 is led by Thrive-LDN, a regional public mental health partnership, with expertise in building coalitions across voluntary organisations, people with lived experience and local/regional government. In order to ensure participatory methods cater to diverse communities which the consortium seeks to support, a range of methodologies will be used. See Table S4 in the Supplementary Materials for selected examples.

We will establish a Scientific and Policy Advisory Board and a Community Insights Panel representing academic and health sciences, policy, implementation, and lived experience, across the UK. The Scientific Policy Advisory Board will have a co-chair with lived experience and also have two members from the Community Insights Panel participating on the Board. To ensure wider inclusion of people outside of the PMH Consortium, we will develop a communications strategy, which will inform website development and other methods for knowledge transfer/exchange. Reflective practice and action learning sets will also run to enable wider and continued learning and development.

*Key outputs.* The key deliverables of this platform will democratise processes around how knowledge is produced, shared, and used. This will include:

1. An embedded survivor researcher methodology that ensures the integration of lived experience in all aspects of the Consortium.
2. Creative methods and outputs for wider engagement, dissemination, and impact, including exhibitions, a children’s book/comic strip, body-maps, animation/short film, and infographics.
3. Three ‘sandpit’ events, which are inclusive of people who have not applied their expertise to population health previously, to innovate intervention development. Sandpit themes will be schools/digital platforms, suicide prevention/digital technologies, and urban planning for population mental health. Participants will be able to apply for competitive funding for projects in these themes. Details of sandpit events will be communicated across the PMH Consortium and to partners. Participants will be selected to ensure a diverse range of perspectives from academic/non-academic and community organisations, as well as representing health, social care and lived experience. Current PMH Consortium members will be excluded from applying. We anticipate that the types of people who may be able to take part in these events (who may not have prior population health expertise) might include people working with children and young people and with schools, people with expertise of digital platforms and technologies, organisations supporting people who have been bereaved by suicide, and people with expertise in urban planning and the built environment (architects, urban planners, structural engineers, and those with expertise in public transport). For each of the sandpits, people with lived experience will inform development of proposed projects and provide input into decision-making processes relating to funding.
4. Policy roundtables will be held, led by the Mental Health Foundation with support from English, Scottish, Welsh, and Northern Ireland Government representatives, to bring together stakeholders, experts, policymakers, and the public to gather feedback, build consensus, and develop actionable recommendations on the PMH Consortium challenges.

#### Cross-Cutting Platform 2: Data, linkages, and causal inference

*Objective.* To leverage novel large-scale and linked data, and refine state-of-the-art natural experiment and causal inference methods, to evaluate the impact of upstream determinants, policy or other major ‘shocks’ (e.g. COVID-19 lockdowns, cost of living crisis, etc) on population mental health inequalities.

*Rationale.* Despite a large evidence base of observational studies indicative of strong associations between mental health and a range of social determinants, including lower income, neighbourhood social adversity, discrimination, isolation, and childhood adversities [3], evaluating the effectiveness of population-level interventions to prevent mental ill-health has proven difficult, as complex interventions designed to address the social determinants of mental health are rarely amenable to randomised controlled trials [4]. Until now, our understanding of causality has been hampered by threats to validity, due to selection bias and unobserved confounding of observational data, impeding our ability to develop effective primary prevention strategies to improve mental health. In addition, whereas advancements have been made in the UK over the last decade in the linkage of health and non-health datasets, further progress has been impeded by restrictions on certain data sources being linked or made publicly available.

*Approach.* This Cross-Cutting Platform will apply novel methods in causal inference, including natural experiment designs [25], to strengthen causal evidence around the social determinants of mental health and the effectiveness of candidate prevention/policy interventions in each Challenge Area. We will:

1. Identify priority research questions on the social determinants of mental health, and how they can be modified to optimise mental health and prevent mental health problems;
2. Identify suitable longitudinal datasets to answer these questions;
3. Apply causal inference, natural experiment or policy evaluation methods to address priority research questions;
4. Support capacity building and knowledge exchange in data, linkages, causal inference methods across each Challenge Area.

*Data.* Various data sources will be explored and used (see Table 2). The UK is a world leader in longitudinal cohorts and repeated cross-sectional surveys with embedded mental health outcomes in large, nationally representative population-based samples. Many of these cohorts are increasingly linked via the UK Longitudinal Linkage Collaboration (UKLLC), permitting largescale analyses on harmonised mental health measures. We will utilise UKLLC linkages to administrative and routine data and review the suitability of other population health data. Beyond the UKLLC, our consortium will also access other available high quality data assets that include relevant mental health outcomes, including other population-based studies (for example, the Catalogue of Mental Health Measures [https://www.cataloguementalhealth.ac.uk/] provides a valuable resource of harmonised UK-based studies), as well as NHS secondary mental health care services linked to administrative data. We will establish the potential for cross-country comparative analyses. Many of the population and nationally representative survey data sources displayed in Table 2 provide valuable information on potential upstream determinants of mental health (education, housing, economic stability, social exclusion, etc.), and though the linkages to health records data, provide potential novel approaches to understanding inequalities in population mental health, which may be less feasible when examined through standard unlinked data sources.

**Table 2:** Examples of candidate UK data sources

Types of Data	Data Sources
Longitudinal population cohorts and repeated population-based cross-sectional surveys	• 1946, 1958, 1970 and Millennium Cohort Study national birth cohorts • UK Household Longitudinal Study • Regional birth cohorts such as the Avon Longitudinal Study of Parents and Children and Born-in-Bradford cohorts.
Population cohort linkages to administrative and routine data (via UKLLC)	• Longitudinal population cohorts as listed above, via UKLLC with linkages to: • Hospital Episode Statistics • Mental Health Services Dataset • NHS Talking Therapies (formerly Improving Access to Psychological Therapies-IAPT) • Office for National Statistics mortality linkages • Department for Work and Pensions linkage • Primary care data
Other population health data	• University College London Million Migrants Study • UKRI Adolescent Health Study • UK Biobank • Department of Health and Social Care Adult Psychiatric Morbidity Surveys
Health records data (includes longitudinal and cross-sectional data)	• Clinical Record Interactive Search databases in partner NHS Mental Health Trusts • Clinical Practice Research Datalink primary care data • Including individual-level data linkages to administrative and other health records data
Cross-country comparative data	• Adolescent Data Platform for Mental Health & the Secure Anonymised Information Linkage databank for Wales • British Heart Foundation-COVID consortia/Open Safely with whole-country primary care linkages to health records/other data for pandemic planning in England • Linked administrative and health records in Northern Ireland.

UKLCC = UK Longitudinal Linkage Collaboration

*Statistical/methodological approaches.* Approaches include Bayesian hierarchical interrupted time series [26] and difference-in-differences models, inverse probability weighting, g-methods, instrumental variable analyses, causal mediation, positive and negative control outcomes, propensity score methods including regression discontinuity, and natural experiments including discordant sibling methods and Mendelian randomisation. Also, evaluation of policy or national programme rollout impacts on health [27], with synthetic controls [28], identification of unintended consequences and ‘spillover’ effects [29], and economic considerations.

*Key outputs.*

1. Increased capacity across the consortium for data, linkage, and training in causal methods in population mental health.
2. Strengthening the causal evidence base in the UK for the effects of social determinants on mental health and illness.
3. Strengthening the causal evidence base in the UK for the effectiveness of population-based prevention strategies, policy interventions and other natural experiments on mental health inequalities, relevant to each Challenge Area.

#### Cross-Cutting Platform 3: Narrowing inequalities

*Objective.* To assess intersectional inequalities to ensure population-based, policy, and systems interventions are equitable.

*Rationale.* National surveys indicate that common mental health conditions are more prevalent among systemically disadvantaged groups, including Black women, adults not in employment, those in receipt of benefits, and those with multiple long-term conditions [2], with strong associations noted between social determinants and mental distress and illness [3, 4]. Health inequalities are avoidable and unjust differences in the experience of health and illness. They are caused by systems of discrimination, powerlessness, and disadvantage that intersect across social class, gender, ethnicity, sexuality, age, and disability. Co-produced research that integrates an intersectional equity lens can reduce these inequities.

*Approach.* We will apply quantitative and qualitative methods to assess intersectional inequalities in population mental health and ensure that interventions and policies do not contribute to a widening of inequalities [29]. Through partnership with the NHS England Advancing Mental Health Equalities Taskforce, we will ensure that our approaches to tackling inequalities are aligned at national and systems levels. With Cross-Cutting Platform 1, we will centre the lived experience of underserved communities, co-producing evidence and solutions.

We will work with Cross-Cutting Platform 2 to apply quantitative methods that enable intersectional assessments of population systems interventions for mental health. A range of advanced methods will be used, such as Latent Class Analysis [30] and Multilevel Analysis of Individual Heterogeneity and Discriminatory Accuracy [31]. We will also visualise geographical and spatial trends in data using ArcGIS StoryMaps, to create interactive maps that convey qualitative place-based narratives.

With Cross-Cutting Platform 1, we will run a peer-led photovoice-based participatory project to engage and capture the experiences of under-served communities, to share narratives and perspectives of those missing from traditional population mental health discourse. Findings will be written up collectively through a workshop with participants and curated as part of an exhibition to amplify people’s voices and engage the broader public.

***Measurement and tools***.

We will use Equality Impact Assessments which, as described above, are a structured and systematic process for evaluating how policies, programmes, and research activities may differentially affect groups defined by protected and socio-demographic characteristics. This process will be used to identify missing voices; reflect on power distributions; assess the quality of involvement of lived experience; critically appraise the evidence base; and assess data availability. It will also enable us to actively consider potential unintended and unanticipated consequences, such as interventions widening inequities, and to adapt our approaches to promote fairness, accessibility, and accountability.

We will also use the Health Inequalities Assessment Toolkit to integrate an intersectional equity lens into our research and involve individuals with lived experience and policy/practice expertise [32]. This tool will allow us to map inequalities, integrate intersectional equity into all research questions, prioritise evidence for action, and ensure accountability in tackling identified inequalities.

*Key outputs*.

1. Evidence-based policy recommendations to reduce health disparities.
2. Interactive mapping of place-based inequities via ArcGIS StoryMaps.
3. Insights on drivers of mental health inequalities across intersecting identities (socio-demographic attributes).
4. Amplified lived experiences through a photovoice exhibition.

#### Cross-Cutting Platform 4: Training and capacity building

*Objective.* To develop interdisciplinary training programmes and educational activities spanning public health, local government, policy, data sciences, population mental health, and lived experience, to support the development of the next generation of leaders in population mental health.

*Rationale.* High fidelity training which provides consistent and coherent approaches to an understanding of mental health remain conspicuously absent in the wider provision of public health training [33, 34].

*Approach.* Drawing on wide-ranging interdisciplinary expertise, Cross-Cutting Platform 4 will tackle this challenge by developing a unique and tailored education and training programme in population mental health. Training will support a range of target audiences, including third sector partners and public health practitioners, university academics, clinicians, early career researchers, survivor researchers, and people with lived experience. We will develop a bespoke programme tailored to meet the needs of the wide range of target groups, to develop knowledge, skills, and understanding around key issues in the field of population mental health. To address the different levels of baseline knowledge, the programme will be carefully designed to ensure inclusivity, with specific bespoke training tailored to the needs of diverse audiences.

*Key outputs.* We will convene a series of in-person, online, and hybrid tutorials, workshops, and presentations. For PMH Consortium members, key outputs will include:

1. Spotlight sessions; themed online one-hour monthly sessions, co-chaired by academic, policy, practice, people with lived experience, and survivor researcher members, with a practical focus related to the Challenge Areas and Cross-Cutting Platforms.
2. Seminar series related to population mental health with tailored tutorials for academic, practitioner, and survivor researchers. The training will be co-developed with Cross-Cutting Platform and Challenge Area leads, including qualitative and quantitative methods and training in lived experience involvement and leadership.
3. Bidirectional and co-produced learning opportunities between survivor researchers and other professional groups in the cluster. Academic/public health/policy methodologists will provide tailored methods training for survivor researchers while survivor researchers will provide tailored training on trauma-informed practice, creative methods, and meaningful lived experience involvement in research.

For individuals and organisations outside of the PMH Consortium, key outputs will include:

1. A Mental Health in All Policies programme (Thrive LDN; [35]) to increase adoption of public health interventions in non-health settings.
2. A novel training suite “New Ways of Working in Population Mental Health” will be co-created across all Challenge Areas and Cross-Cutting Platforms. This will include recurrent training workshops and videos via accessible platforms such as FutureLearn.
3. In-person training will be advertised through the National Centre for Research Methods.
4. We will explore the potential to adapt training into accredited postgraduate courses/modules and other freely available resources, drawing upon a mapping and critical appraisal of existing provision around population mental health.

#### Challenge Area 1: Children and young people’s mental health

*Objective.* To develop mental health intelligence systems to support school leaders, local decision-makers, and central government in planning and evaluating equitable interventions for prevention of mental ill-health in children and young people (CYP).

*Rationale.* Three quarters of mental health conditions are apparent by age 24 [5], with rises in certain conditions in adolescents and young people following the pandemic [36]. Over the past decade, the UK government and devolved administrations have made substantial commitments to enhancing mental health and prevention of mental ill-health, early detection, and intervention for CYP, including investment in school-based support for mental health. However, unmet mental health needs among CYP remain high and inequitably distributed across geographic regions and sociodemographic strata. School or local-level data resources that can support estimation of CYP population mental health needs and inform targeted interventions are urgently needed, but remain limited.

*Approach.* Using existing innovative linked whole-country national pupil-level data between education and health in England (the Mental Health Services Data Set nested in ECHILD), and in Wales (Secure Anonymised Information Linkage databank), we will undertake foundational work to examine whole nation longitudinal patterns of CYP mental health service activity (emergency, primary, and secondary care) nested for schools, to produce routine estimates and temporal trends of ‘known’ mental health needs. We will build on our expertise to tackle data quality issues [37].

Next, we will compare estimates of school-level mental health needs to other local or regional data sources including linked school-level mental health registries (e.g. South London National Pupil Database-Clinical Record Interactive Search) [37] and regional cross-sectional health surveys (e.g., Student Health and Wellbeing Survey in Wales) [38] and annual school census wellbeing data in Birmingham [39]. These comparisons will inform understanding of the relationships underpinning data on service availability and use, unmet need across population strata to aid planning and investigation of equitably distributed CYP preventative programmes such as Mental Health Support Teams, whole-school bullying programmes, or school-based parenting training programmes. To support universal accessibility, we will investigate how population estimates are altered in CYP who are neurodiverse or racially minoritised.

Finally, we will work with Challenge Area 2 to examine longer-term impacts on adverse outcomes like self-harm/suicide. Through our partnerships with Cross-Cutting Platform 1, we will assess how these data can be used to engage stakeholders from schools, national decision-makers, and the public.

*Main goals (to be examined at national*,* regional*,* and school level).*

1. Building capacity and capabilities to use linked routinely captured mental health services data and education data to describe CYP population mental health.
2. Exploring the levels of agreement between routinely captured data and self-report mental health data in relation to potential social determinants.
3. Demonstrating the strengths and weaknesses of using these data for informing population mental health local and national planning.

#### Challenge Area 2: Prevention of suicide and self-harm

*Objective.* To evaluate the effectiveness of public health interventions impacting suicide and self-harm inequalities in the short, medium, and long-term.

*Rationale.* Approximately six thousand people die by suicide annually in the UK [40]. The economic cost of each suicide was estimated to be approximately £1.46 million in 2022 [41], and the effects on those bereaved by suicide are immeasurable. There is emerging evidence for divergence in suicide risk by region, socioeconomic indices, and ethnicity [42]. These inequalities could be exacerbated by the current cost of living crisis and other post-pandemic societal stressors. Therefore population-wide monitoring of trends, and effective public health interventions that reduce risk across all sociodemographic groups, must be prioritised.

*Approach.* Internationally, there is evidence for a range of population-level interventions to reduce suicide risk; for example, pack size restrictions for over-the-counter analgesics [43]. Some candidate intra-UK policy differences for ‘natural experiment’ investigations include free prescriptions for psychotropic medications, in Northern Ireland, Scotland, and Wales, but not England, and minimum pricing per unit of alcohol in Scotland and Wales, but not England. We shall consider two types of interventions: those with a primary intention to prevent self-harm and/or suicide (‘Type 1’) versus those with a different intended impact (‘Type 2’); for instance, initiatives to ameliorate known risk factors for suicidal behaviours such as childhood adversity, domestic violence, gambling, and loneliness.

Broadly, we will:

1. Conduct an international umbrella review to synthesise evidence from systematic reviews of means restriction initiatives in the field of suicide and self-harm prevention;
2. Work with Cross-Cutting Platform 2 to determine which population-based datasets and inter-source linkages are available for tackling the most relevant and pertinent research questions, and to overcome key methodological challenges;
3. Consult extensively with a diverse range of stakeholders, through Cross-Cutting Platform 1;
4. Work closely with Challenge Area 1 because rising self-harm incidence in adolescents and young adults [36] is now a UK public health priority.

*Data.* We will utilise datasets such as the Secure Anonymised Information Linkage Databank (Wales), the Greater Manchester Secure Data Environment, and the Greater London Thrive LDN Real Time Surveillance System for suspected suicides. Due to the rarity of suicide in the general population, it will be difficult to discern impacts of policy change on suicide rates at local authority level. This will apply especially to subtle perturbations in risk that may arise following a Type 2 intervention, the primary purpose of which was not to prevent suicide. Thus, we shall focus most of our investigations of suicide on national level comparisons (across UK nations), whilst examining non-fatal self-harm at a more granular level (e.g., local authority level).

*Key outputs.*

1. An overview of how population-level initiatives aimed specifically at reducing self-harm and suicide differentially impact socioeconomic and demographic groups, to support policy decisions on national and location-specific measures. We will utilise methodologies such as umbrella reviews of published systematic reviews that have synthesised evidence reported from population-based empirical studies.
2. Empirical evidence for the impact of national public health policies. This will inform decisions about implementing and continuing such policies.

#### Challenge Area 3: Multiple long-term conditions

*Objective.* To evaluate policies, public health interventions, and group-level approaches impacting mental health inequalities in people with multiple long-term conditions (MLTCs).

*Rationale.* Other than suicide, most premature deaths in people with mental health conditions are from preventable physical causes [44]. Around 14 million people in England have MLTCs [12]. Mental and physical health have important bi-directional associations; our work has found that MLTCs exacerbate each other, with a spiral of worsening physical, emotional, cognitive, and social functioning [45]. MLTCs elevate the risk of developing mental distress and ill health via modifiable psychosocial, environmental, and behavioural pathways [44].

*Approach.* We will work with Cross-Cutting Platform 1 to identify relevant policy or public health interventions, alongside identifying suitable and comprehensive datasets (e.g. before-after intervention introduction, or cross-country comparisons). The focus would be on a syndemics framework, aiming to evaluate multi-domain interventions/policies spanning upstream and downstream drivers (e.g., individual, community, system). This could include assessing national rollout of population-based programmes for physical health to evaluate impacts on mental health [27]. Other potential interventions include: devolution of powers to local government for health and social care [28], physical activity campaigns, alcohol minimum pricing, the introduction of ‘Integrated Wellness Services’ (community services providing integrated care for wider determinants of health), the Supporting Families programme (systems approach to support vulnerable families to overcome multiple challenges to improve physical and mental health), and extending the age of retirement. In parallel, we will review existing data on published natural experiments in these areas, conduct systematic reviews, and, if appropriate, meta-analyses. Having identified plausible areas for exploration, we will work with Cross-Cutting Platform 2 to identify datasets and develop the most appropriate methods (e.g. interrupted time series analyses) to assess the impact of policy/public health interventions on mental health outcomes in MLTC populations.

*Data.* Potential data sources which have been identified include whole population linked data for England, including primary care, NHS Talking Therapies (formerly IAPT) and the Mental Health Services Data Set (British Heart Foundation-COVID Consortium), the Secure Anonymised Information Linkage databank for Wales, and Honest Broker Service and Northern Ireland Statistics and Research Agency health linked data for Northern Ireland. Further sources could include pooled and harmonised UK cohorts’ data linked to electronic health records (e.g., Understanding Society, Birth cohorts (1958, 1970, 1990, and 2000 s). We will triangulate the evidence across these diverse yet complementary data sources to inform and guide preventative interventions aiming to reduce the population burden of mental ill-health in the context of MLTCs (and vice versa), adopting a health inequalities lens.

*Key outputs.*

1. Establish the infrastructure to facilitate comparative effectiveness research aiming to address population mental health priorities within the context of MLTCs.
2. Identify individual and system-level policies and interventions that may aggravate or ameliorate existing mental health outcomes disparities across different MLTC populations.
3. Develop real-world guidelines and recommendations for public health policies aiming to address growing mental health inequalities in the context of MLTCs.

### Dissemination and impact

The PMH Consortium will contribute to the prevention of mental health issues, with personal, economic, social, and health benefits in the UK. We will achieve impact through establishing new ways of working, leading to new channels of communication and engagement, and new mechanisms for change. Our PMH Consortium will deliver outputs and intermediate outcomes (see Table 3) that will translate into long-term, scalable, and sustainable impact in the post-project sustainability phase.

**Table 3:** Knowledge exchange, transfer, and impact through the Population Mental Health (PMH) Consortium

Activity/Output	Likely reach/beneficiaries	Measurable outcomes	Longer-term impact	
Academic papers, chapters, reports, and presentations	Wider research community (national and international)	New evidence related to population prevention for mental health and research methods	Prevention of mental health issues with economic benefits/cost savings and improved quality of life	
‘Sandpit’ events	Events engaging PMH Consortium members and non-members outside of PMH	Interdisciplinary collaborations; Pump-primed projects	New projects, approaches, and methods for prevention of mental health issues	
Policy roundtables	Events including academics and policymakers	Actionable recommendations for research and policy	Evidence-based policy, commissioning, and legislation	
Creative outputs (films, website, blogs, infographics, comics, art)	Wide community reach via social media	Knowledge transfer with public and for people outside Consortium to link to our work	Raised public awareness of mental health and the work of the Consortium, and reduced stigma of mental health problems.	
Stakeholder engagement events	Stakeholders, community members, and lived experience experts	Knowledge exchange for Consortium stakeholders	Raised awareness and improved engagement nationally	
Infrastructure for children and young people’s (CYP) mental health intelligence		Schools, government, researchers, CYP, and families	Validated measures for CYP to inform assessments of mental health preventative interventions	Better prevention and improved CYP mental health, providing social, health, and cost-saving benefits
Regional and cross-country studies on suicide and self-harm		Researchers, policymakers, impacted people, and families	Policies and public health evidence on suicide and self-harm prevention	Prevention of suicide and self-harm, providing large health, social, and cost-saving benefits
Evidence to improve mental health in people with multiple long-term conditions (MLTCs)		Researchers, health and social care, people with MLTCs, and families	Population-level interventions for improved mental health in people with MLTCs	Benefits to people with MLTCs and their families, as well as health, social care, and cost saving improvements.
Academic conference	Event including academics, lived experience experts, and policymakers nationally	Knowledge exchange and dissemination for academics	Diffusion of research, leading to new methods and knowledge	
Public curated exhibitions	Consortium members and the public	Opportunities for public to connect with work	Awareness and change in public perceptions of population mental health	
Data management, opensource code	Data science community	Accelerated dissemination of data-based methods and approaches, new collaborations	Faster innovation and improved application of data to health challenges; efficient working	
Capacity building	Early Career Researchers, Consortium members, and the wider community	Critical appraisal of existing gaps in training/education; Knowledge exchange across silos; Training of ECRs, survivor researchers, wider community	Next generation of researchers developed, change in ways of working across silos	
Development of additional projects	Consortium members, collaborators, and Early Career Researchers	New grants/PhD studentships	Longer term sustainability of Consortium and capacity building	

CYP = children and young people’s, MLTCs = multiple long-term conditions, PMH = population mental health

### Ethics and risks

Ethics applications will be made on a project-by-project basis within the PMH Consortium at leading institutions, respectively. A data management plan is in place which details how all research data will be handled throughout the lifecycle of a project, from acquisition to archival. A risk assessment workshop held in the foundational phase identified risks around delivery, impact, collaboration, accountability, lack of vision, finances, staffing, and political influence. We are mitigating these risks in the Consortium through: introducing a formal highlight report mechanism and using an appropriate sequencing/dependencies design; determining evaluation tools and regularity of their use; finalising collaboration agreements; developing a communications plan; organising events which align with the availability of key members, to bring people together; providing early examples of good collaborative practice and outputs; stating and restating the vision for the Consortium; introducing a finance reporting mechanism and determining how finance will be managed within each organisation; monitoring outputs and relationships across time; tracking changing views of funders and politicians; and seeking additional funding on the back of successful activities.

## Discussion and conclusion

There is an urgent need for population-level interventions that address upstream determinants contributing to mental distress, ill health, and inequities in the UK. The siloed nature of current approaches [11] and focus on treatment over prevention [46, 47] has greatly limited improvements in population mental health. At present, there is insufficient evidence in the UK to guide effective and timely policymaking for prevention. Through our PMH Consortium, we will progress this underdeveloped field, address current gaps in knowledge, and develop new ways of working to identify and communicate effective upstream systems approaches for improving population mental health.

Although randomised controlled trials are the ‘gold standard’ for identifying causal effects and are commonly relied upon in evidence-based policy development [48], it is often not feasible to apply these study designs and methodologies to population mental health interventions. Population-level mental health interventions that target upstream determinants are often distal to outcomes, involve complex causal pathways, and are shaped by multiple intersecting social and structural factors. Because these interventions are not amenable to randomisation, a range of alternative methods are required to assess their impacts and build the evidence base [4, 49–51]. As described across the Cross-Cutting Platforms and Challenge Areas, the remit of the PMH Consortium will be to overcome these barriers by harnessing the potential of large-scale linked UK data and applying high quality methods, in partnership with people with lived experience and policy stakeholders, to evaluate the impact of policies which may have benefits for population mental health.

### Challenges we face

The PMH Consortium is a large programme of work, bringing together diverse sectors, disciplines, and stakeholders across the UK. We anticipate operational issues which are common to all large and highly interdisciplinary research projects, which will require new ways of working and thoughtful coordination and structuring. Building trust within the communities the Consortium serves, by ensuring meaningful involvement and trauma-informed approaches, will be essential for improving outcomes and creating lasting impact. Similarly, nurturing relationships between researchers and policy-makers/decision-makers will be key for ensuring evidence can be effectively communicated and translated into policies to improve population mental health.

It is possible that some datasets or linked data are not publicly available, or are highly restricted, which may inhibit or delay the Consortium’s research objectives. Determining and implementing mechanisms through which processes of data acquisition, management, and analysis can be harmonised will be critical. Ensuring accessible and transparent ways of presenting data to all stakeholders, particularly those from the communities involved, will also be key to demonstrating the benefits of the research for population mental health.

### Strengths in our approach

Systems change can only be achieved with strong and integrated partnerships. Our leadership team, comprising researchers, policymakers, and people with lived experience, reflects this. The PMH Consortium Co-Directors bring research, academic, and clinical expertise (JD) and public sector, political engagement, policy expertise, and lived experience of mental health and neurodiversity (DB). Our interdisciplinary Consortium will bring together leading UK governmental agencies, wider public sector agencies, people with lived experience, voluntary organisations, and academic partners, spanning disciplines from science through to the arts. The integrated Consortium structure will ensure multifaceted dialogue between stakeholders, to support meaningful impact.

The ultimate goal of the PMH Consortium is to develop a model for population mental health research and policy translation that is both scalable and sustainable, ensuring continued impact and viability beyond the initial four years/phases. We believe that our described approach is novel and offers advantages which will help to overcome existing challenges and barriers in this space. We are currently in the foundational phase of the Consortium’s life cycle and this paper offers transparency in the aims, objectives, and approach of our Consortium, enabling new individuals and groups to identify and collaborate on the work.

## Supplementary Information

Supplementary Material 1

## Data Availability

No datasets were generated or analysed during the current study.

## Abbreviations

CYP: Children and young people
IAPT: Improving Access to Psychological Therapies
MLTC: Multiple long-term conditions
NHS: National Health Service
PHIU: Population Health Improvement UK
PMH: Population Mental Health
UKLC: UK Longitudinal Linkage Collaboration
