# Supplementary material for: Towards a new model of population mental health research and policy translation in the UK: establishing a National consortium
Source: Int J Ment Health Syst. 2026 May 2;20:17. doi: 10.1186/s13033-026-00703-2 (PMC13285060; doi:10.1186/s13033-026-00703-2)
Supplement: Supplementary file 1 — Supplementary Material 1 [file 13033_2026_703_MOESM1_ESM.docx]

**Towards a new model of population mental health research and policy translation in the UK: Establishing a National Consortium**

**Supplementary Material**

**Table of Contents**

[**Table S1:** GRIPP2 reporting checklist of patient and public involvement in research 2](#_Toc220876701)

[**Table S2:** Working definitions of lived experience in the Population Mental Health Consortium 3](#_Toc220876702)

[**Table S3:** Survivor Researcher Methodology Definitions for the Population Mental Health Consortium 5](#_Toc220876703)

[**Table S4:** Participatory design methodologies underpinning consortium activities, selected examples 6](#_Toc220876704)

## **Table S1: GRIPP2 reporting checklist of patient and public involvement in research**

| **Section and topic** | **Item** | **Reported on page number** |
| --- | --- | --- |
| 1: Aim | Report the aim of PPI in the study | Page 10 under section “2.4 Ethical, effective, and equitable ways of working: Centring lived experience and ensuring equality, diversity, and inclusion”. |
| 2: Methods | Provide a clear description of the methods used for PPI in the study | Pages 10-12 provides a description of methods to be used. |
| 3: Study results | Outcomes—Report the results of PPI in the study, including both positive and negative outcomes | Intended results of PPI are reported on pages 15-16 and will include an embedded survivor researcher methodology, and Artist Survivor Researcher-led outputs. PPI will shape sandpit events, policy roundtables (pages 15-16), training/capacity building (pages 21-22), research (page 11), and knowledge exchange and impact (Table 4). |
| 4: Discussion and conclusions | Outcomes—Comment on the extent to which PPI influenced the study overall. Describe positive and negative effects | Page 30 in Discussion “3.1 Challenges we face” and “3.2. Strengths in our approach”. |
| 5: Reflections/critical perspective | Comment critically on the study, reflecting on the things that went well and those that did not, so others can learn from this experience | Page 30 provides a description of potential strengths/ limitations. |

*PPI = patient and public involvement. From Staniszewska S, et al. GRIPP2 reporting checklists: tools to improve reporting of patient and public involvement in research BMJ 2017; 358 :j3453 doi:10.1136/bmj.j3453*

| **Table S2: Working definitions of lived experience in the Population Mental Health Consortium** The following definitions are shared as *living definitions* — open to feedback, discussion, and refinement by the Consortium’s Community Insights Panel, over time. They were created with lived experience experts.  It is important to recognise that Community Insights Panel members bring forms of knowledge that no one else can — knowledge that is critical to population mental health research. Lived experience, especially when shaped by collective resistance or activism, offers insight that is not simply anecdotal or contextual, but valuable and legitimate in its own right.  We also acknowledge the importance of collective knowledge held by communities — often rooted in activism — which extends beyond individual experience. These definitions aim to honour and reflect that complexity, and we welcome ongoing input to strengthen and evolve them.  **Definition of Community Insights**  Community insights are a form of knowledge that deepens our understanding of local needs and issues, gathered through community engagement and localised activities. These insights emerge from relationship-building with individuals and communities, reflecting their lived experiences.  Community insights can serve as either a mirror, reflecting our own experiences, or a window into the lives of others. Whether we relate to these insights personally or learn from others, we recognise the complexity of human experiences. This helps us develop empathy and enriches our understanding of population mental health needs. As a result, community insights provide valuable evidence alongside data-driven research, offering important context to people’s lived experiences.  **Definition of Lived Experience**  In this context, community insights will focus on the following lived experiences:  Having experienced mental distress or mental health issues, particularly;   - in childhood or adolescence (Challenge 1); - including self-harm, suicidal ideation, or loss through suicide (Challenge 2); - and/or in conjunction with other long-term conditions, including physical health issues (Challenge 3); - Having experienced structural disadvantage, discrimination, marginalisation, inequities, injustice, neglect, abuse, or violence of any kind (interpersonal and/or systemic), which either caused, worsened, were compounded by, or were connected in some way to the mental distress or mental health issues experienced. - Having experienced race-based trauma, where racial discrimination, marginalisation, or systemic racism has directly or indirectly contributed to the mental distress or mental health issues experienced. - Having experienced intersecting forms of discrimination or disadvantage (such as age, disability, gender reassignment, marriage and civil partnership, pregnancy and maternity, race, religion or belief, sex, and sexual orientation), which have compounded or exacerbated mental distress or mental health issues. - We will also consider people who have lived experience of disadvantage, inequality, hardship, poverty etc. who haven’t had experiences of mental health challenges. This is to explore whether this will tell us something about resilience of well-being that might be equally important to our understanding of public mental health.   *Perspectives drawn from lived experience provide retrospective insights not only on mental health struggles but also on risk, protective, and preventative factors; providing key insights on what should be considered for mental health prevention. To understand and address mental health issues and their bidirectional relationship with inequalities, we must understand these from the perspectives of those most affected and ensure that a diverse range of experiences are considered.* |
| --- |

| **Table S3: Survivor Researcher Methodology Definitions for the Population Mental Health Consortium** Survivor research is founded on the value of first-person, experiential knowledge (Russo, 2012). In survivor research, researchers reflect on points of connection and disconnection within and across survivors’ experiences, creating a form of ‘deep experiential knowledge’ (Noorani et al, 2019) which offers new ways of understanding phenomena. A fundamental principle of survivor research is that it should be additive rather than extractive; this means that participants feel valued rather than exploited and that the research in some way contributes to the work and goals of our communities. Survivor research serves epistemic justice by centring non-hierarchical, decolonial ways of conducting research by and for the communities affected (Sweeney et al, 2009; Gastaldo et al, 2018). As survivor research challenges ‘what we think we know’ (Faulkner, 2017, p. 501), it leads to the production of a different kind of knowledge that cannot be produced through mainstream approaches (Sweeney et al., 2009).  **References:**  Russo, J. (2012). ‘Survivor-Controlled Research: A New Foundation for Thinking about Psychiatry and Mental Health’, Forum Qualitative Social Forum: Qualitative Social Research, 13(1)  Noorani, T., Karlsson, M., Borkman, T. (2019). ‘Deep experiential knowledge: reflections from mutual aid groups for evidence‐based practice’, Evid Policy, 15(2): pp. 217‐234.  Sweeney, A. et al (2009). This Is Survivor Research. Monmouth: PCCS Books.  Gastaldo, D. et al. (2018). ‘Body-Map Storytelling as a Health Research Methodology: Blurred Lines Crearing Clear Pictures’, Forum Qualitative Social Research, 19(2).  Faulkner, A. (2017). Survivor research and Mad Studies: the role and value of experiential knowledge in mental health research. Disability & Society, 32(4), 500-520. |
| --- |

## **Table S4: Participatory design methodologies underpinning consortium activities, selected examples**

| **Activity type** | **Stages where participation invited** | **Co-creation methodologies** | **Format** | **Participants** |
| --- | --- | --- | --- | --- |
| Design of research studies | Members from the Community Insights Panel are involved in initial study plans, interpretation of findings, dissemination. Artist survivor researchers join/ participate in project meetings. | Advisory group meetings between community insights panel and researchers. Involvement of artist survivor researchers in research project team meetings. | Small group presentations and consultative workshops, 1:1 meetings and small group meetings. | Researchers sharing work with up to 10 community insight panel members, ~6 artist survivor researchers embedded within research project meetings. |
| Artist survivor researcher-led outputs | Artist survivor researchers will lead projects and will join/ contribute to consortium meetings, workshops and other events. | Artist survivor research-led projects, including creative arts methodologies, which will be informed by consortium research themes. | Outputs to be presented as curated art exhibitions, and development of a written methodology on embedding artist survivor work. | 500+ attendees (outside of the consortium/ general public) engaging with survivor-led art at curated exhibitions. |
| Policy roundtables | Roundtable events bringing together policy makers from across devolved government departments, people with lived experience and researchers to inform development of research questions/ dissemination of findings. | Large participatory roundtable events with smaller breakout groups and larger plenary discussions. | 50-70 attendees at facilitated workshop events, including smaller groups for discussion. | 50-70 participants comprising consortium researchers and people with lived experience and policy stakeholders representing local, regional and national government. |
| Sandpit events | Sandpit events inviting participation of people who have not previously worked in population mental health improvement to pitch research ideas for small funds. | Facilitated workshop events to support people to broker collaborations in population mental health research for seed funding. | Attendees from a range of backgrounds (community organisations, academics, public health, people with lived experience) at one-day events, facilitated to broker new collaborations. | 30-40 attendees who are outside of the population mental health consortium and with little prior or no knowledge of population mental health, but with relevant interdisciplinary expertise, (e.g. urban built environments, schools, digital platforms and technologies). People with lived experience involved with judging applications for funding. |

**Table example adapted from Hidalgo et al. 2019*
